# Increase in the prevalence of mutations associated with sulfadoxine–pyrimethamine resistance in *Plasmodium falciparum* isolates collected from early to late pregnancy in Nanoro, Burkina Faso

**DOI:** 10.1186/s12936-017-1831-y

**Published:** 2017-04-28

**Authors:** Esmée Ruizendaal, Marc C. Tahita, Ronald B. Geskus, Inge Versteeg, Susana Scott, Umberto d’Alessandro, Palpouguini Lompo, Karim Derra, Maminata Traore-Coulibaly, Menno D. de Jong, Henk D. F. H. Schallig, Halidou Tinto, Petra F. Mens

**Affiliations:** 1 Department of Medical Microbiology, Academic Medical Centre, Amsterdam, The Netherlands; 2 Institut de Recherche en Sciences de la Santé-Unité de Recherche Clinique de Nanoro, (IRSS-URCN), Nanoro, Burkina Faso; 3 Department of Clinical Epidemiology, Biostatistic and Bioinformatics, Academic Medical Centre, Amsterdam, The Netherlands; 4 Oxford University Clinical Research Unit, Wellcome Trust Major Overseas Programme, Ho Chi Minh City, Viet Nam; 5 Koninklijk Instituut voor de Tropen, Amsterdam, The Netherlands; 6 Department of Infectious Disease Epidemiology, London School of Hygiene and Tropical Medicine, London, UK; 7 Disease Control and Elimination, Medical Research Council Unit, Fajara, Gambia; 8 Department of Disease Control, Faculty of Infectious and Tropical Diseases, London School of Hygiene and Tropical Medicine, London, UK

**Keywords:** *Plasmodium falciparum*, Pregnancy, Sulfadoxine–pyrimethamine, Resistance, Mutations

## Abstract

**Background:**

Pregnant women are a high-risk group for *Plasmodium falciparum* infections, which may result in maternal anaemia and low birth weight newborns, among other adverse birth outcomes. Intermittent preventive treatment with sulfadoxine–pyrimethamine during pregnancy (IPTp-SP) is widely implemented to prevent these negative effects of malaria. However, resistance against SP by *P. falciparum* may decrease efficacy of IPTp-SP. Combinations of point mutations in the *dhps* (codons A437, K540) and *dhfr* genes (codons N51, C59, S108) of *P. falciparum* are associated with SP resistance. In this study the prevalence of SP resistance mutations was determined among *P. falciparum* found in pregnant women and the general population (GP) from Nanoro, Burkina Faso and the association of IPTp-SP dosing and other variables with mutations was studied.

**Methods:**

Blood spots on filter papers were collected from pregnant women at their first antenatal care visit (ANC booking) and at delivery, from an ongoing trial and from the GP in a cross-sectional survey. The *dhps* and *dhfr* genes were amplified by nested PCR and products were sequenced to identify mutations conferring resistance (ANC booking, *n* = 400; delivery, *n* = 223; GP, *n* = 400). Prevalence was estimated with generalized estimating equations and for multivariate analyses mixed effects logistic regression was used.

**Results:**

The prevalence of the triple *dhfr* mutation was high, and significantly higher in the GP and at delivery than at ANC booking, but it did not affect birth weight. Furthermore, quintuple mutations (triple *dhfr* and double *dhps* mutations) were found for the first time in Burkina Faso. IPTp-SP did not significantly affect the occurrence of any of the mutations, but high transmission season was associated with increased mutation prevalence in delivery samples. It is unclear why the prevalence of mutations was higher in the GP than in pregnant women at ANC booking.

**Conclusion:**

The high number of mutants and the presence of quintuple mutants in Burkina Faso confirm concerns about the efficacy of IPTp-SP in the near future. Other drug combinations to tackle malaria in pregnancy should, therefore, be explored. An increase in mutation prevalence due to IPTp-SP dosing could not be confirmed.

**Electronic supplementary material:**

The online version of this article (doi:10.1186/s12936-017-1831-y) contains supplementary material, which is available to authorized users.

## Background

Malaria remains an important cause of morbidity and mortality worldwide, in particular in sub-Saharan Africa (SSA) [1]. Certain populations, in particular children under five years of age and pregnant women, are more at risk of developing clinical malaria. Young children generally experience multiple episodes of clinical (sometimes severe) malaria before acquiring partial immunity against this disease [2, 3]. In areas where malaria transmission is stable, women have acquired partial immunity, but they remain vulnerable to malaria when they get pregnant due to specific surface antigens on *Plasmodium falciparum*-infected erythrocytes that are almost exclusively presented by the malaria parasite during pregnancy [4]. This leaves in particular primigravidae, who are exposed to these antigens for the first time, more vulnerable to malaria infection. Women who are more frequently exposed to these pregnancy-associated malaria antigens, like multigravidae, can acquire a certain degree of immunity and are therefore less severely affected by malaria in pregnancy [5–8]. Malaria in pregnancy may result in maternal anaemia, low birth weight, pre-term birth, still birth, and infant and maternal deaths [9, 10]. Most SSA countries have implemented preventive anti-malarial treatment schedules to protect the high-risk groups, such as intermittent preventive treatment in infants (IPTi) or pregnant women (IPTp). In IPTp, pregnant women in their second and third trimester of pregnancy living in moderate to high malaria transmission areas are offered sulfadoxine–pyrimethamine (SP) at each antenatal care (ANC) visit, irrespective of clinical malaria symptoms or diagnostic test results [11]. SP is a suitable drug for this preventive regimen because of its excellent safety profile during pregnancy and its prophylactic efficacy [12, 13]. However, although IPTp-SP has greatly reduced morbidity in pregnant women and their offspring, there are growing concerns about its waning efficacy due to an increase in parasite resistance against SP [14, 15]. In particular, in certain parts of East Africa resistance against SP is extremely high necessitating alternative preventive strategies [16, 17]. In West Africa resistance against SP is rising, but IPTp-SP is still considered efficacious [18]. Nevertheless, monitoring SP resistance is of utmost importance to ensure timely changes in policy. Emergence of SP resistance can be monitored by determining the prevalence of mutations in two genes of *P. falciparum*, dihydropteroate synthase (*dhps*) and dihydrofolate reductase (*dhfr*), associated with resistance against sulfadoxine and pyrimethamine, respectively. Accumulation of single point mutations in these genes leads to increasing SP resistance, particularly the combined triple *dhfr* (N51, C59, S108) and double *dhps* (A437, K540) mutations (quintuple mutant) [19].

In Burkina Faso, SP was used as first-line malaria treatment until 2005 but has since been used only for IPTp-SP and IPTi [20]. Mutations in the *dhps* and *dhfr* gene are nevertheless still increasing (around 10–30% increase from 2009 to 2012) [15]. This may be due to the continued use of SP in IPTi and IPTp programmes that are still causing selection of SP-resistant parasites. In the present study, carried out in central Western Burkina Faso, the prevalence of the main SP resistance related mutations (*dhps* S436, A437, K540 and *dhfr* N51, C59, S108 codons) was determined in pregnant women at ANC booking, at delivery and in the general population (GP) [15, 21–23]. It was hypothesized that the prevalence of SP resistance mutations would increase over pregnancy due to exposure to IPTp-SP, thus reaching the highest prevalence at delivery. The comparison with the GP was made to investigate whether pregnant women unexposed to IPTp-SP (at ANC booking) show comparable mutation prevalence to the GP. Furthermore, this study aimed to identify factors other than IPTp-SP that are associated with increased risk of finding mutant parasites.

## Methods

### COSMIC: study population and procedures

This study was done in the framework of a cluster-randomized intervention trial (Community-based scheduled screening and treatment of malaria in pregnancy for improved maternal and infant health: a cluster-randomized trial, or COSMIC, registration numbers ISRCTN372259296 Current Controlled Trials and NCT01941564 clinicaltrials.gov). The study was situated in the Nanoro health centre catchment area, which is a rural area located 85 km northwest of Ouagadougou. The area is highly endemic for malaria with the peak transmission season occurring towards the end of the rainy season, which lasts from June until October. The COSMIC trial has been described elsewhere [24]. Briefly, the intervention trial investigated the effect of malaria screening and treatment of pregnant women by community health workers at village level on placental malaria and low birth weight. The study was implemented in 30 villages, 15 control and 15 intervention villages. Women from both control and intervention villages were enrolled in the study at their first ANC visit at which they received standard care, including IPTp-SP according to national guidelines. Women were subsequently followed up until delivery. At each ANC visit and at delivery a blood slide was collected and blood was spotted on Whatman 3MM filter paper or 1MM filter paper (spots of at least 12-mm diameter), depending on availability. In intervention villages, pregnant women also received monthly malaria screening at village level with rapid diagnostic test (RDT, SD Bioline Ag-Pf). In case of a positive RDT artemisinin-based combination therapy was given (artemether–lumefantrine). At delivery, the birth weight of the newborn was collected for all participants. ANC booking and delivery samples collected from May 2014 until September 2015 were tested for *P. falciparum* by real-time PCR. All *P. falciparum*-positive delivery samples and 400 randomly selected positive ANC booking samples were included in the analyses of SP resistance markers.

### Cross-sectional survey: study population and procedures

Samples from the GP were collected from March until May 2015 in the Nanoro health centre catchment area in the same villages as the COSMIC study (*n* = 30). Field workers and nurses were trained for 1 week on malaria, its clinical symptoms, RDT diagnosis, blood spot collection, treatment of malaria, and in obtaining informed consent. In each household two members were randomly selected for the survey. In each village 20 households were randomly selected from the database of the Health and Demographic Surveillance System. For 205 selected households, the residents were not present at the time of visit and these were therefore replaced by 205 other randomly selected households. Participants had to be ≥6 months of age. Pregnant women or participants that reported SP use in the past 2 weeks were not enrolled. After informed consent was obtained, medical history and clinical symptoms or signs of malaria were recorded by the field workers or nurses. The nurses subsequently performed a RDT (SD Bioline Ag-Pf) and collected blood spots of at least 12-mm diameter on Whatman 3MM filter paper. RDT-positive participants were treated according to national guidelines. All filter paper samples were tested for *P. falciparum* by real-time PCR, 400 positive samples were randomly selected to study the SP resistance markers.

### Filter paper processing and DNA extraction

Blood spots on filter paper were air dried, put in sealable bags with silica and transported on the same day from the field to the laboratory (Unité de Recherche Clinique de Nanoro-URCN). Filter paper samples were stored at ambient temperature until shipment to the laboratory in The Netherlands. From each filter paper, one blood spot was punched out using Acu-punch skin biopsy punchers (Acuderm^®^ Inc, USA) and transferred into a 5-mL polystyrene tube. Subsequently 2 ml of EasyMAG lysis buffer (*bioMérieux*, Marcy-l’Étoile, France) was added to the tubes after which they were put on a roller bank for 30 min for thorough lysis. After lysis, the fluid was transferred to EasyMAG vessels and magnetic bead silica were added to each well according to manufacturer’s instructions. DNA was extracted with DBS 1.0 protocol in the NucliSENS EasyMAG. Positive controls (blood spots from diluted *P. falciparum* 3D7 or FCR3 in vitro culture) and negative controls (blood spots of uninfected erythrocytes obtained from the Dutch Blood Bank) were included. Samples of extracted DNA were stored at −20 °C until further use.

### Real-time PCR

*Plasmodium falciparum* DNA was detected by real-time PCR, as described previously, with minor adjustments in primer concentrations and probe sequence [25–27]. Per reaction, 2.5 µl of DNA, 5 mM MgCl_2_, 2.5 µl of 10× PCR Buffer, 0.125 µl of HotStarTaq DNA Polymerase, 0.25 mM of each dNTP, 0.4 µM of each primer and 0.1 µM of FAM-labelled probe (′5-aacaattggagggcaagg-3′) were used. All PCR mix reagents were ordered from Qiagen (Hilden, Germany) and all primers from Biolegio (Nijmegen, The Netherlands). In each plate a dilution series of *P. falciparum* FCR3 culture was included (tenfold dilution steps with highest value 10^4^ parasites/µl and a lowest value of 1 parasites/µl) as well as positive and negative DNA extraction controls and Milli-Q water. The dilution series were used to make an estimation of the parasitaemia. Reactions were run on BioRad CFX real-time PCR machine with the following settings: initial denaturation 95 °C for 10 min, 40 cycles of 95 °C for 60 s and 60 °C for 20 s. Results were analysed using Bio-Rad CFX manager software (version 3.1).

### Nested PCR and sequencing

Nested PCR was used to amplify the *dhfr* and *dhps* gene regions covering the codons of interest. Nest 1 primers were described in Duraisingh et al. [28]. Nest 2 primers were located just inwards of nest 1 primers (Table 1). All primers were ordered from Biolegio (Nijmegen, The Netherlands). For nest 1 *dhfr* PCR, 2.5 µl of DNA, 1 mM MgCl_2_, 2.5 µl of 10× PCR Buffer, 0.125 µl of HotStarTaq DNA Polymerase, 0.25 mM of each dNTP, and 0.4 µM of each primer were used per reaction. For nest 1 *dhps* the same reagents were used except that 2 mM of MgCl_2_ was used instead of 1 mM. For nest 2, both PCRs used 0.5 µl of nest 1 product, 2 mM MgCl_2_, 2.5 µl of 10× PCR Buffer, 0.125 µl of HotStarTaq DNA Polymerase, 0.25 mM of each dNTP and 0.4 µM of each primer. All PCR mix ingredients were ordered from Qiagen (Hilden, Germany). Nest 1 PCR settings were: initial denaturation at 94 °C for 10 min, 28 cycles of 94 °C 1 min, 58 °C 1 min, 74 °C 1 min. Nest 2 PCR settings were: initial denaturation at 94 °C for 10 min, 40 cycles of 94 °C 1 min, 58 °C 1 min, 74 °C 1 min. Positive (*P. falciparum* D10 genomic DNA, MRA-201G, MR4, ATCC^®^ Manassas Virginia, USA) and negative controls (uninfected erythrocytes) were included in each PCR. Products were run on a 1% agarose gel. Clear bands of the correct size were sent for sequencing with the corresponding nest 2 primers (Macrogen, Amsterdam, The Netherlands). For *dhps,* two additional reverse primers were used for a total of four sequencing reactions (Table 1). In this way a homopolymer region in the middle of the *dhps* PCR product, that caused polymerase slippage, was circumvented. Results were analysed with Geneious 8.1.8 using a *P. falciparum* 3D7 reference genome. Base pair calling was manually confirmed in both sequencing directions for each sample (or the two reverse directions for *dhps* K540). Heterozygote results had to be confirmed in both sequencing directions to be qualified as a mixed infection. Mixed infections were considered mutants in the initial analyses.

**Table 1:** Primer sequences used for nested PCR and sequencing of *Plasmodium falciparum dhfr* and *dhps* genes

Primer name	Sequences
Nest 1 forward *dhfr*	5′-TTTATGATGGAACAAGTCTGC-3′
Nest 1 reverse *dhfr*	5′-AGTATATACATCGCTAACAGA-3′
Nest 2 forward *dhfr*	5′-TCTGCGACGTTTTCGATATTT-3′
Nest 2 reverse *dhfr*	5′-CTCATTTTCATTTATTTCTGGA-3′
Nest 1 forward *dhps*	5′-AACCTAAACGTGCTGTTCAA-3′
Nest 1 reverse *dhps*	5′-AATTGTGTGATTTGTCCACAA-3′
Nest 2 forward *dhps*	5′-CCTAAACGTGCTGTTCAAAGAA-3′
Nest 2 reverse *dhps*	5′-TTGTTCATCATGTAATTTTTGTTGTG-3′
Nest 2 reverse2 *dhps*	5′-CAATACTTATAATTGGTTTCGCATCA-3′ (sequencing only)
Nest 2 reverse 540 *dhps*	5′-TTCGCAAATCCTAATCCAATATC-3′ (sequencing only)

### Statistical analysis

Data were analysed using Stata 14.1. Descriptive statistics were used to identify the characteristics of the three study populations (ANC booking, delivery, GP). Clustering was present at village level and also because of repeated measurements (some women were included in both the ANC samples and delivery samples). In each of the three study populations, mutation prevalence with 95% confidence intervals was estimated by generalized estimating equations with village as panel variable in order to obtain a population-averaged effect. However, to enable multiple levels in some of the univariate and multivariate models explaining the mutation prevalence in the data sets, for example, repeated measurements in pregnant women who are clustered in villages, mixed effects logistic regressions were used for all explanatory models. Type of visit (ANC versus delivery and ANC versus GP), IPTp-SP doses or IPTp-SP dose in past 30 days, and other variables, such as age, parasitaemia, artemether–lumefantrine treatment, gravidity, and season were used as fixed effects in univariate mixed effects logistic regressions for each mutation of interest. The high transmission season was defined as the period between June and November. All variables of the univariate analyses were also included in the multivariate model, except parasitaemia because this was considered an intermediate variable for the effect of IPTp-SP doses on the outcome. In the multivariate model for pregnant women, an interaction effect was added for season versus visit, denoted seasonXvisit, and for gravidity versus visit, denoted gravidityXvisit. For both pregnant women and the ANC/GP model, an interaction effect for age and visit was included, denoted ageXvisit. Age was centred at 25 years (median of pregnant women) and reported per 10-year increase. For analyses of each of the three study populations and for ANC and GP samples combined, only village was included as random intercept. For analyses of the ANC and delivery combined sample set, participant identifier was nested within village as random intercepts. Household was not included as random variable because no information was available about overlapping households between pregnant women and the GP. After initial analyses in which mixed infections were considered mutants, the same analyses were conducted for pure mutants versus mixed and wild type infections combined.

Lastly, univariate and multivariate analyses were performed on the association between the triple *dhfr* mutation and low birth weight (<2500 g) in delivery samples, using village as a random effect and covariates IPTp-SP dosing, season, gravidity, age and artemether-lumefantrine exposure as fixed effects. The confidence intervals and *p* values of all conducted analyses are shown, unadjusted for multiple comparisons.

## Results

### COSMIC: mutation prevalence and predicting factors

**Fig. 1:**
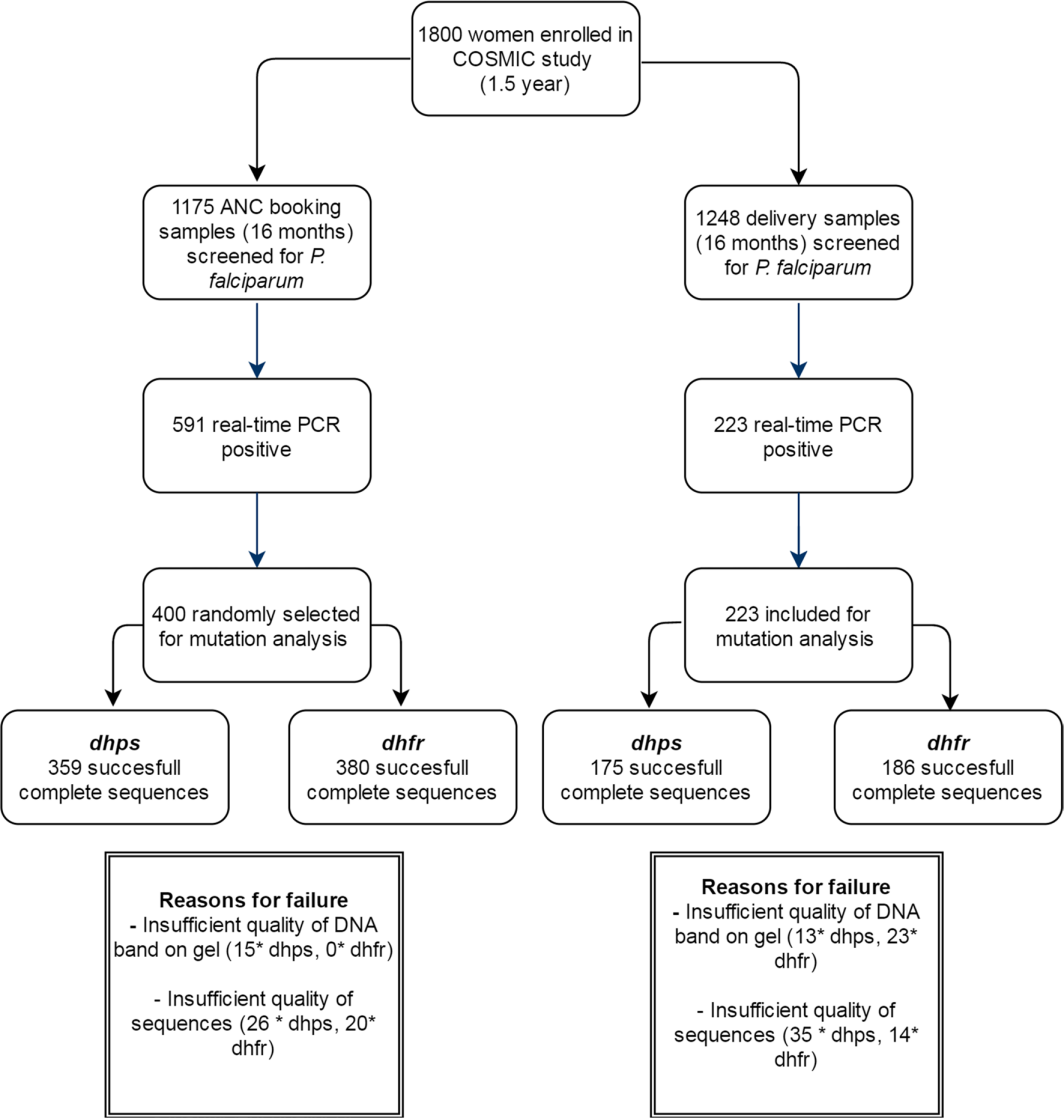
Flowchart of the longitudinal survey of SP resistance mutations in pregnant women

About half of the samples collected at ANC booking (591/1175, 50.3%) were tested positive for *P. falciparum* by real-time PCR while this was 17.9% (223/1248) at delivery. A random selection of 400 *P. falciparum*-positive ANC samples and all 223 positive delivery samples were included for mutation analyses by sequencing (Fig. 1). Characteristics of the participants for each study population are presented in Table 2. Almost all women received at least one dose of IPTp-SP. 57.4% received the former recommended minimum of two doses of IPTp-SP and 15.3% received four or more doses of IPTp-SP. Parasite density was generally lower at delivery than at ANC, with a median of 6 and 30 parasites/µl, respectively.

**Table 2:** Characteristics of study participants in the longitudinal and cross-sectional survey

Characteristics	Longitudinal survey		Cross-sectional survey
	ANC booking *(n* = 400)	Delivery (*n* = 223)	GP (*n* = 400)
Age in years, median (IQR)	24 (20–28.5)	25 (19–30)	10 (6–25)
Female, %	100	100	52
Gravidity			
Primigravidae, %	25.8	28.3	NA
Secundigravidae, %	20.0	12.6	NA
Multigravidae, %	54.3	59.2	NA
Number of IPTp-SP doses, %			
0	NA	0.5	NA
1	NA	15.3	NA
2	NA	57.4	NA
3	NA	11.7	NA
4+	NA	15.3	NA
Temperature °C, mean (IQR)	36.7 (36.4–37.0)	36.8 (36.5–37.1)	36.9 (36.6–37.0)
Fever, temperature >37.5 °C, %	1.5	10.0	1.5
Parasitaemia p/µl, median (IQR)	30 (7–147)	6 (1–102)	22 (4–141)
Parasitaemia sub-groups p/µl, %			
0–10	32.8	54.7	39.3
10–100	37.8	20.2	31.5
100–1000	25.5	15.3	23.8
1000–10,000	4	8.1	5
>10,000	0	1.8	0.5
Birth weight in grams, mean (SD)	NA	2950 (520)	NA
Low birth weight (<2500 g), %	NA	16.7	NA

Sequence results for the three codons associated with SP resistance in the *dhps* gene (S436, A437, K540) could be obtained from 359 (89.8%) ANC samples and 175 (78.5%) delivery samples. For the three *dhfr* codons (N51, C59, S108) results were available for 380 (95.0%) ANC and 186 (83.4%) delivery samples. High proportions of mutant parasites were found for all codons and the triple *dhfr* mutation in both study populations, but not for *dhps* K540 (Table 3 and Additional file 1: Table S1). The K540 mutation was detected in two delivery samples. As the *dhps* A437 and the triple *dhfr* mutation were also detected in these samples, parasites in these samples can thus be considered quintuple mutants. In univariate mixed effects logistic regression analyses delivery samples showed significantly higher prevalence of mutations compared with ANC samples for *dhps* A437 (*p* = 0.037), *dhfr* C59 (*p* = 0.013), *dhfr* S108 (*p* = 0.016) and triple *dhfr* mutations (*p* = 0.029) and a trend for higher *dhfr* N51 (*p* = 0.069) mutations (Additional file 2: Table S2). Univariate analyses also revealed a significant positive association between gravidity and all *dhfr* mutations and between rainy season and all *dhfr* mutations for delivery samples (*p* < 0.05 and *p* < 0.01, respectively). Increased age was associated with a higher risk of *dhfr* N51 (*p* = 0.004) and triple *dhfr* mutations (*p* = 0.005) in ANC samples, while treatment with artemether–lumefantrine was associated with a higher risk of the same mutations in delivery samples (*p* = 0.001 and *p* = 0.002, respectively). No significant associations were seen for parasitaemia or IPTp-SP doses (Additional file 2: Table S2).

**Table 3:** Prevalence of *Plasmodium falciparum* double *dhps,* triple *dhfr* and quintuple mutations in ANC, delivery and GP samples

	ANC booking		Delivery		GP	
	% mutant (95% CI)	*N*	% mutant (95% CI)	*N*	% mutant (95% CI)	*N*
Double *dhps* mutation	0	359	1.1 (0.3–4.4)	175	1.1 (0.4–2.8)	352
Triple *dhfr* mutation	61.1 (56.2–65.2)	380	70.5 (63.9–76.5)	182	73.9 (67.9–78.9)	355
Quintuple mutation	0	353	1.2 (0.3–4.6)	168	0.9 (0.3–2.6)	336

Univariate analyses for the outcome of low birth weight at delivery did not show an association with the triple *dhfr* mutation, but IPTp-SP doses, age and gravidity were all protective of low birth weight (*p* = 0.003, *p* = 0.046 and *p* = 0.004, respectively) (Additional file 3: Table S3).

In multivariate analyses (Table 4) for ANC and delivery samples, there was no significant association between number of IPTp-SP doses and prevalence of SP resistance mutations (*p* > 0.05). The inclusion of SP exposure in the past 30 days in the model, instead of the number of SP doses, resulted in larger odds ratios (OR) but the associations were still not significant (Additional file 4: Table S4). The interaction term between age and visit was not significant, but a significantly lower prevalence of *dhfr* N51 (*p* = 0.010) and triple *dhfr* (*p* = 0.010) mutations was seen for older age at ANC booking. This was seen to a lesser extent (*p* = 0.075 and *p* = 0.051), in delivery samples. Higher gravidity resulted in increased prevalence of mutations in delivery samples (significant for *dhfr* N51, *p* = 0.042, and triple *dhfr, p* = 0.024, mutations). Delivery samples showed increased prevalence of *dhfr* mutations in high transmission season compared with low transmission season (*p* = 0.007, *p* = 0.015, *p* = 0.016, *p* = 0.002 for *dhfr* N51, *dhfr* C59, *dhfr* S108 and triple *dhfr*, respectively). For ANC samples there was no such seasonal difference. For the *dhps* gene there were no significant variables in multivariate analyses except for decreased prevalence of *dhps* A437 mutations (*p* = 0.047) with increasing artemether–lumefantrine doses.

**Table 4:** Multivariate mixed-effects logistic regression for *dhfr* and *dhps* mutations in ANC and delivery samples (pregnant women)

Fixed effect(s)	*dhfr*																*dhps*							
	N51				C59				S108				triple *dhfr*				S436				A437			
	OR	[95% CI]		*p*	OR	[95% CI]		*p*	OR	[95% CI]		*p*	OR	[95% CI]		*p*	OR	[95% CI]		*p*	OR	[95% CI]		*p*
Age (10 years)	0.29	0.12	0.74	*0.010*	0.46	0.17	1.29	0.133	0.47	0.16	1.38	0.170	0.39	0.18	0.80	*0.010*	2.58	0.85	2.94	0.145	0.71	0.29	1.70	0.441
Gravidity	1.25	0.97	1.60	0.087	1.12	0.84	1.50	0.432	1.17	0.85	1.60	0.328	1.16	0.94	1.44	0.165	0.75	0.54	1.06	0.100	1.05	0.81	1.36	0.720
Season^a^	1.08	0.68	1.72	0.747	1.07	0.61	1.87	0.805	1.05	0.58	1.90	0.880	1.05	0.69	1.60	0.825	1.58	0.85	2.94	0.145	0.73	0.43	1.22	0.226
IPTp-SP doses	1.09	0.72	1.65	0.672	1.70	0.87	3.34	0.121	2.25	0.94	5.43	0.070	1.04	0.72	1.52	0.819	1.02	0.65	1.60	0.930	1.44	0.81	2.59	0.218
AL	0.64	0.37	1.11	0.109	0.72	0.35	1.47	0.364	0.84	0.38	1.84	0.658	0.67	0.43	1.06	0.090	0.89	0.51	1.55	0.679	0.57	0.33	0.99	*0.047*
Visit^b^	0.23	0.03	1.84	0.167	0.09	0.00	1.58	0.099	0.04	0.00	1.39	0.076	0.22	0.03	1.44	0.114	0.25	0.02	2.93	0.267	0.83	0.07	9.42	0.880
SeasonXvisit	4.32	1.31	14.25	*0.016*	7.56	1.30	43.95	*0.024*	11.30	1.41	90.44	*0.022*	4.17	1.51	11.55	*0.006*	3.00	0.11	85.88	0.520	2.60	0.65	10.44	0.179
Visit^b^ in high transmission season	1.00	0.16	6.31	0.998	0.66	0.06	7.29	0.736	0.47	0.03	7.56	0.593	0.93	0.17	5.00	0.929	0.38	0.04	3.66	0.401	2.15	0.23	20.41	0.504
Season^a^ in Del samples	4.66	1.52	14.27	*0.007*	8.11	1.50	43.76	*0.015*	11.83	1.59	87.93	*0.016*	4.37	1.73	11.07	*0.002*	2.44	0.70	8.53	0.161	1.88	0.52	6.85	0.337
AgeXvisit	1.00	0.23	4.34	0.995	1.34	0.21	8.36	0.754	1.66	0.20	1343	0.636	0.85	0.22	3.25	0.814	0.44	0.07	2.76	0.378	1.06	0.19	6.11	0.945
Age in Del samples	0.30	0.08	1.13	0.075	0.62	0.12	3.24	0.567	0.78	0..11	5.28	0.797	0.33	0.11	1.01	0.051	1.10	0.26	4.73	0.898	0.75	0.17	3.41	0.712
GravidityXvisit	1.26	0.79	2.02	0.337	1.26	0.69	2.29	0.455	1.29	0.64	2.58	0.480	1.30	0.85	1.98	0.219	1.33	0.76	2.33	0.319	0.90	0.53	1.53	0.706
Gravidity in Del samples	1.57	1.02	2.42	*0.042*	1.41	0.81	2.45	0.221	1.50	0.78	2.91	0.226	1.52	1.06	2.18	*0.024*	1.00	0.64	1.56	0.995	0.95	0.60	1.50	0.817

Odds ratios (OR) with 95% CI and *p* values are presented (*p* values <0.05 in italics)

*AL* artemether–lumefantrine therapy, *Del* delivery

^a^Low transmission season = 0, high transmission season = 1

^b^ANC booking = 0, Delivery = 1; age centred at 25 years

Multivariate analysis for the effect of the triple *dhfr* mutation on the outcome of low birth weight was conducted for delivery samples including IPTp-SP doses, gravidity, age, season and artemether–lumefantrine therapy. IPTp-SP and gravidity were both protective against low birth weight (*p* = 0.008 and *p* = 0.010, respectively), but none of the other variables was significantly associated with low birth weight (including the triple *dhfr* mutation) (Additional file 5: Table S5).

### Cross-sectional survey: mutation prevalence and predicting factors

**Fig. 2:**
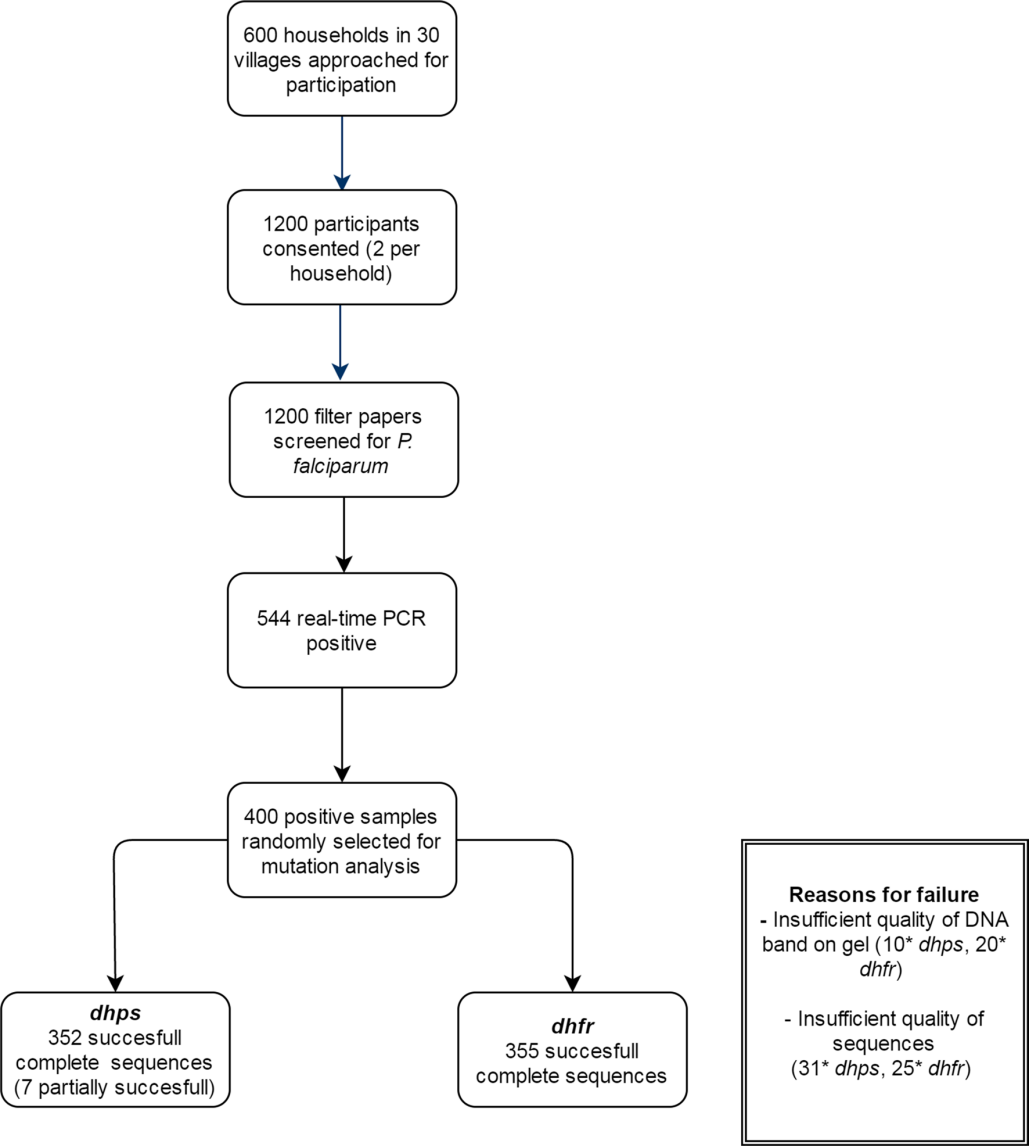
Flowchart of the cross-sectional survey of SP resistance mutations in the general population

Out of 1200 GP participants, 45.3% (544) were tested positive for *P. falciparum* by real-time PCR. 400 positive samples were randomly selected for mutation analyses (Fig. 2). Characteristics of included participants are shown in Table 2. The median age of participants was 10 years and 52% were female. The median parasite density was 22 p/µl.

Sequence results could be obtained from 352 (88.0%) *dhps* samples and 355 (88.8%) *dhfr* samples. There were six samples with good quality sequence results for only the *dhps* K540 codon and one sample for only the *dhps* S436, A437 codons. All tested codons showed high proportions of mutants, except for *dhps* K540 which was found in only four of the 358 samples (Table 3). Three of these were quintuple mutants, the fourth had missing data for the *dhfr* sequence. The proportions of mutants in GP samples were higher compared with ANC samples, but were similar to delivery samples. In univariate analysis GP samples showed significantly higher prevalences of *dhfr* N51, C59, S108, triple *dhfr* mutations, and *dhps* S436 mutations (all *p* < 0.001) compared with ANC samples. Delivery and GP samples were not significantly different (Additional file 2: Table S2).

In multivariate analyses (Table 5) for GP and ANC samples, older age was again associated with decreased prevalence of *dhfr* N51 (*p* = 0.004) and triple *dhfr* (*p* = 0.005) mutations for pregnant women at ANC booking, but there was no association of age with mutation prevalence in GP samples. For participants of the same age, GP samples showed significantly higher prevalence of all *dhfr* (*p* < 0.01) and *dhps* S436 (*p* = 0.014) mutations than ANC samples. However, in a sensitivity analysis in which age was restricted to 15–45 years in the GP group (range in pregnant women), GP showed a similar trend in age as ANC samples. GP still showed significantly higher *dhfr* N51 (*p* = 0.006) and triple *dhfr* mutation prevalence (*p* = 0.025) (Additional file 6: Table S6). Season was no significant predictor for any of the mutations.

**Table 5:** Multivariate mixed-effects logistic regression for *dhfr* and *dhps* mutations in ANC (pregnant women) and GP samples

Fixed effect(s)	*dhfr*																*dhps*							
	N51				C59				S108				triple *dhfr*				S436				A437			
	OR	[95% CI]		*p*	OR	[95% CI]		*p*	OR	[95% CI]		*p*	OR	[95% CI]		*p*	OR	[95% CI]		*p*	OR	[95% CI]		*p*
Age	0.59	0.41	0.85	*0.004*	0.69	0.48	1.01	0.056	0.78	0.53	1.15	0.210	0.60	0.42	0.86	*0.005*	1.04	0.70	2.33	0.113	0.82	0.54	1.23	0.334
Season^a^	1.07	0.70	1.65	0.758	1.06	0.68	1.67	0.795	1.04	0.65	1.65	0.877	1.05	0.69	1.60	0.821	1.46	0.91	2.33	0.113	0.73	0.43	1.22	0.225
Visit^b^	2.02	1.33	3.06	*0.001*	1.87	1.21	2.89	*0.005*	2.06	1.30	3.25	*0.002*	1.89	1.26	2.84	*0.002*	1.70	1.11	2.58	*0.014*	1.14	0.70	1.87	0.602
VisitXage	1.70	1.15	2.52	*0.008*	1.30	0.87	1.95	0.206	1.11	0.73	1.69	0.628	1.70	1.16	2.50	*0.007*	0.83	0.55	1.25	0.369	1.12	0.72	1.75	0.612
Age in GP samples	1.01	0.86	1.18	0.931	0.90	0.77	1.06	0.210	0.87	0.73	1.03	0.098	1.03	0.89	1.19	0.723	1.46	0.91	2.33	0.113	0.92	0.78	1.08	0.293

Odds ratios (OR) with 95% CI and *p* values are presented (*p* values <0.05 in italic)

^a^low transmission season = 0, high transmission season = 1

^b^ANC booking = 0, GP = 1; age centred at 25 years

### Mutant versus mixed and wild type infections in ANC, delivery and GP samples

**Fig. 3:**
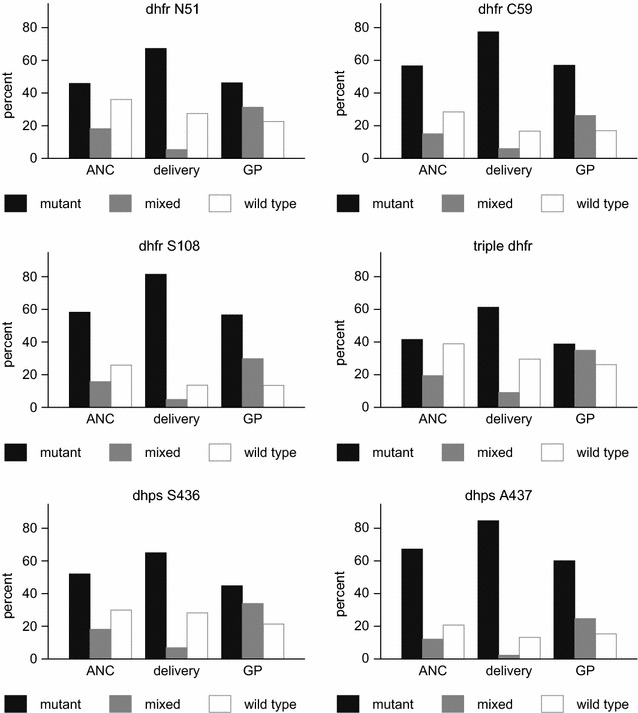
Prevalence of mutant, mixed and wild type alleles in ANC, delivery and GP samples

Stratifying the sequence results in mutant, mixed or wild type codons revealed that the difference in mutation prevalence between ANC and delivery samples was mainly related to a difference in pure mutants, while the difference between ANC and GP samples was mainly related to a difference in mixed infections (Fig. 3). In univariate analyses for pure mutants versus mixed and wild type infections, delivery samples had significantly increased prevalence of all mutations compared with both ANC and GP samples (all *dhfr* mutations, *p* ≤ 0.001; triple *dhfr* mutation*, p* ≤ 0.001; *dhps* S436 mutation, *p* = 0.014 for ANC/delivery and *p* ≤ 0.001 for GP/delivery; and *dhps* A437 mutation, *p* ≤ 0.001). In contrast, GP and ANC samples showed comparable prevalence of resistance mutations (Additional file 7: Table S7). In multivariate analyses for pregnant women, high transmission season resulted once again in increased *dhfr* mutation prevalence in delivery samples. Also higher gravidity remained associated with increased prevalence of *dhfr* N51 and triple *dhfr* mutations in delivery samples. The trend of a negative association between age and mutation prevalence in ANC and delivery samples remained, but this was only significant for the triple *dhfr* mutation in delivery samples (Additional file 8: Table S8). In the ANC/GP model none of the variables was significant, except for an association between older age and increased prevalence of mutations in codons *dhfr* N51, triple *dhfr* and *dhps* A437 (*p* = 0.046, *p* = 0.014 and *p* = 0.010, respectively) in GP samples (Additional file 9: Table S9).

## Discussion

Overall the prevalence of SP resistance related mutations was high among Burkinabe pregnant women and the non-pregnant population for all three *dhfr* codons studied (N51 ≥ 63.9%, C59 ≥ 71.6%, S108 ≥ 74.2%, triple *dhfr* ≥ 61.1%) and for two *dhps* codons (S436 ≥ 70.9% and A437 ≥ 79.4%). However, both delivery and GP samples showed significantly higher prevalence of *dhfr* and either S436 or A437 *dhps* mutations than ANC samples. In 2010 in the same region, the prevalence of SP resistance mutations was considerably lower, especially for *dhps* codon A437 (34.2%), *dhfr* codon N51 (12.2%) and the *dhfr* triple mutation (11.4%) [23]. This suggests a substantial increase in mutation prevalence in a 5-year period. However, the previous study used a different mutation analysis method, restriction fragment length polymorphism, which could also explain the differences. Recent studies in other regions of Burkina Faso reported prevalences of mutant parasites that were more similar to those observed here. In 2010–2011, among pregnant women at ANC booking, the prevalence of mutations in *dhfr* N51, C59, S108 codons (49.1, 50.4 and 57.8%, respectively) was lower but the prevalence of *dhps* S436 and A437 mutations (81.2 and 75.2%, respectively) was similar to the currently presented results [22]. Between 2009 and 2011 Geiger et al. [15] showed an upward trend for prevalence of *dhfr* and *dhps* mutations in samples from the general population reaching comparable or higher levels to the prevalences reported here (from graph: S436 ± 88% A437 ± 80%, N51 ± 80%, C59 ± 90%, S108 ± 90%).

Another important finding was the presence of *dhps* K540 mutations in six samples, of which five were considered quintuple mutants. This is the first time quintuple mutants are found in Burkina Faso. In 2002, the *dhps* K540 mutation was found in one study in Burkina Faso, but these were not quintuple mutants [29]. Despite the importance of this finding, for the current situation in which triple *dhfr* mutations are common but the quintuple mutation is rare, IPTp-SP is still expected to be efficacious [18]. This is also substantiated by the finding that the triple *dhfr* mutation was not associated with low birth weight, while an increasing number of IPTp-SP doses did protect malaria infected women at delivery from giving birth to low birth weight babies.

Although the prevalence of mutations (codons *dhps* A437 and *dhfr* C59, S108 and the triple *dhfr* mutation) was higher at delivery than at ANC booking, there was no evidence of a significant association between IPTp-SP doses and selection of resistant parasites. Because all but one woman had received at least one dose of IPTp-SP at delivery, a binary variable of IPTp-SP exposure could not be included in the regression model. SP exposure in the past 30 days was also included as a binary variable, to account for time between SP exposure and sampling, but the results were still not significant. Previous studies on the selection of SP resistance mutations by IPTp-SP show contradicting results, with some showing no effect of IPTp-SP on the mutation prevalence [21, 30–32], while others do find higher prevalence in women reporting SP use [16, 33, 34]. These contradicting results could be due to a combination of different epidemiological settings and methodological approaches.

Evidence for a higher prevalence of *dhfr* mutations in low versus high transmission season had been observed in the general population in Burkina Faso by Geiger et al. [15] and was therefore included in the analyses. However, in the current study an opposite seasonal effect of higher *dhfr* mutation prevalence in the high transmission season in delivery samples and no seasonal effect for ANC samples was found. Increased *dhfr* mutations in the high transmission season have been described before in non-pregnant individuals in Indonesia, but the authors related this phenomenon to increased drug exposure, while this is not the case in the present study in which IPTp-SP was given year round [35]. Therefore, it seems that elevated transmission intensity increases the survival chances for mutant parasites, yet only at the end of pregnancy, since this phenomenon was not observed in ANC samples. The question is how this happens. Increased transmission likely increases the prevalence of mixed infections, which was indeed seen for delivery samples. This can result in within-host competition between malaria strains leading to suppression of one or both (or multiple) strains, as has been described before for *P. falciparum* in humans [16, 36] and *Plasmodium chabaudi* in mice [37]. Competition may result from limitations in available resources or can be immune mediated [38]. However, in the presence of drug exposure (like SP in delivery samples), a phenomenon called competitive facilitation could occur, in which drug-resistant *P. falciparum* suddenly have increased growth rates due to clearance of wild type strains after chemotherapy [16, 37]. Thus the increased competition together with (recent) SP exposure could be an explanation for the selection of mutant parasites in delivery samples during high transmission season.

Older age was significantly associated with decreased *dhfr* N51 and triple *dhfr* mutation prevalence in ANC samples and non-significantly in delivery samples. Associations between age and *dhfr* mutation prevalence in pregnant women have not been frequently described in previous literature, but there were two reports that found no association [39, 40]. The same accounts for gravidity being positively associated with mutation prevalence in delivery samples. There has been one report that found a similar positive association [40], but in another study there was no effect of gravidity on mutation prevalence [39].

Strikingly, the resistance levels found in GP samples were comparable to samples collected at delivery and significantly different (for all *dhfr* mutations and *dhps* S436 mutations) from samples collected at ANC booking. SP exposure is unlikely to be the cause of the higher mutation prevalence in GP samples; there was no seasonal malaria chemoprevention (SMC) programme with SP in the area and only infants at age two, three and four months received IPTi (thus excluded from the study). Only one person from the GP reported use of cotrimoxazole (not included in analyses), a drug that shares the same drug targets of sulfadoxine and pyrimethamine [41]. Noteworthy however is the fact that the difference in resistance between GP and ANC samples was mainly found in mixed infections (considered mutants), whereas the difference between delivery and ANC samples was mainly found in pure mutants. The increase in mutation prevalence due to mixed infections in GP samples could not be explained by season of sampling as this was corrected for in multivariate analyses. However, age distribution was also a noticeable difference; GP participants were mostly children <15 years of age, whereas pregnant women were aged 15–45 years. In children, age has been shown to be inversely related to *dhfr* S108 and quintuple mutation prevalence [42, 43]. Nevertheless, the difference in prevalence of *dhfr* N51 and triple *dhfr* mutations compared with ANC samples remained even if only GP participants aged 15–45 years were included in the model. Therefore, other unknown factors that differ between pregnant and non-pregnant individuals must be involved in infection dynamics of wild type and mutant parasites.

The differences seen for *dhps* mutations between the three visits were less pronounced than for *dhfr*. Furthermore, almost no variables included in the multivariate models were significantly associated with mutation prevalence in the *dhps* gene. It is likely that selection of mutations on the *dhps* gene is independent of selection of *dhfr* mutations and that it is driven by other factors [44].

There are some limitations to this study. First, samples from the general population were collected in a cross-sectional survey at the end of the dry season. As discussed above, season had an effect on delivery samples, but did not seem to affect the differences found between ANC and GP samples. However, future evaluations of SP resistance mutations should consider performing year round surveys or repeated cross-sectional surveys in both low and high transmission season for the general population. Additionally, it is possible that pregnant women and the GP are too diverse in infection dynamics and immunity to compare these groups for mutation prevalence. It would therefore be interesting to evaluate SP mutation prevalence in *P. falciparum* samples from non-pregnant women of child-bearing age versus pregnant women.

Another limitation is that there were quite some sequencing failures (5–22%), more so for delivery samples than GP and ANC samples. Failures were mainly due to low parasite density (median parasitaemia was 1 p/µl for failed *dhfr* and 2 p/µl for failed *dhps* sequences) and this was more common in delivery samples (Table 2). However, it is unlikely that these failures have led to a large bias in the resistance profiles. Parasitaemia was not associated with mutation prevalence in ANC samples and in delivery samples there was a trend of higher prevalence of mutations in samples with a low parasite density, so if the failed delivery samples would have been successfully sequenced they may even have increased the difference in mutation prevalence between early and late pregnancy. The GP samples showed an opposite trend of increased prevalence of mutations in samples with a low parasite density, but the difference in number of sequencing failures and parasitaemia with ANC samples was much smaller, thus the risk of bias is small.

Lastly, the current study lacks information on additional mutation sites in the *dhfr* and *dhps* genes, such as *dhps* A581, A613 and *dhfr* I164. In particular the *dhps* A581 mutation in combination with the quintuple mutation, also known as the sextuple mutant, has been associated with reduced efficacy of IPTp-SP [45–47]. Nevertheless, because the quintuple mutation was scarcely found in the study population, monitoring additional *dhps* mutations is important for the future but is unlikely to have impacted the current study results. Furthermore, the *dhps* A581 and *dhfr* I164 mutation have not been previously found in Burkina Faso or its neighbouring countries [14, 22].

## Conclusion

This study shows that that resistance mutation profiles are dynamic and dependent on the study population (e.g., pregnant women in early and late pregnancy and also non-pregnant individuals) and transmission setting. Overall a high prevalence of *dhfr* N51, C59 and S108 and *dhps* S436 and A437 mutations is found in the study area. A combination of transmission intensity and SP exposure is a plausible cause of increased mutation prevalence in high transmission season in delivery samples. Nevertheless, it seems that selection of resistant mutants is a complicated process in which most likely aspects like epidemiology, immunity and infection dynamics and drug exposure are involved. These aspects are subject to change, as the adherence to the new WHO policy of offering IPTp-SP at each ANC visit is getting better and SMC with SP is being widely implemented, thus impacting the selection and spread of SP resistance mutations. This fact and the new finding of quintuple mutants circulating in the area therefore asks for thorough surveillance of emerging resistance against SP. Although currently IPTp-SP is still likely to remain effective in the study area, a search for alternative strategies is justified.

## Additional files

**Additional file 1.** Prevalence of *P. falciparum dhps* and *dhfr* mutations in ANC, delivery and GP samples.

**Additional file 2.** Univariate mixed-effects logistic regression for *dhfr* and *dhps* mutations in *P. falciparum* positive samples.

**Additional file 3.** Univariate mixed-effects logistic regression for low birth weight in *P. falciparum* positive women at delivery.

**Additional file 4.** Multivariate mixed-effects logistic regression for *dhfr* and *dhps* mutations in pregnant women (recent IPTp-SP use).

**Additional file 5.** Multivariate mixed-effects logistic regression for low birth weight in *P. falciparum* positive women at delivery.

**Additional file 6.** Multivariate mixed-effects logistic regression for *dhfr* and *dhps* mutations in ANC and GP samples (sensitivity analysis).

**Additional file 7.** Univariate mixed effects logistic regression for *dhfr* and *dhps* gene mutations (pure mutants versus wild type/mixed).

**Additional file 8.** Multivariate mixed effects logistic regression for *dhfr* and *dhps* gene mutations in pregnant women (pure mutants versus wild type/mixed).

**Additional file 9.** Multivariate mixed effects logistic regression for *dhfr* and *dhps* gene mutations in pregnant women sampled at ANC booking and the GP (pure mutants versus wild type/mixed).

## Abbreviations

IPTi: intermittent preventive treatment for infants
IPTp: intermittent preventive treatment for pregnant women
SP: sulfadoxine–pyrimethamine
*dhps*: dihydropteroate synthase
*dhfr*: dihydrofolate reductase
ANC: antenatal care visit
GP: general population
SMC: seasonal malaria chemoprevention
