# Supplementary material for: Increase in the prevalence of mutations associated with sulfadoxine–pyrimethamine resistance in Plasmodium falciparum isolates collected from early to late pregnancy in Nanoro, Burkina Faso
Source: Malar J. 2017 Apr 28;16:179. doi: 10.1186/s12936-017-1831-y (PMC5410088; doi:10.1186/s12936-017-1831-y)
Supplement: Supplementary file 1 — Additional file 1. Prevalence of P. falciparum dhps and dhfr mutations in ANC, delivery and GP samples. [file 12936_2017_1831_MOESM1_ESM.pdf]

Table S1. Prevalence of *P. falciparum dhps* and *dhfr* mutations in ANC, delivery and GP samples.

dhps						
	ANC booking		Delivery		GP	
Codons	% mutant (95% CI)	*n*	% mutant (95% CI)	*n*	% mutant (95% CI)	*n*
S436	70.3 (64.3 – 75.4)	359	72.1 (65.5 – 77.9)	175	78.9 (74.6 – 82.5)	353
A437	78.4 (75.4 – 81.3)	359	86.8 (59.9 – 91.5)	175	84.8 (80.2 – 88.6)	353
K540	0	359	1.1 (0.3 – 4.4)	175	1.1 (0.4 – 2.7)	358
double *dhps*	0	359	1.1 (0.3 – 4.4)	175	1.1 (0.4 – 2.8)	352
dhfr						
N51	63.9 (59.1 – 68.6)	380	72.7 (66.2 – 78.4)	182	77.6 (71.7 – 82.5)	355
C59	71.7 (67.0 – 75.8)	380	83.2 (76.7 – 88.1)	182	83.2 (78.4 – 87.0)	355
S108	74.3 (69.6 – 78.4)	380	86.4 (80.4 – 90.9)	182	86.5 (82.2 – 90.8)	355
triple *dhfr*	61.1 (56.2 – 65.2)	380	70.5 (63.9 – 76.5)	182	73.9 (67.9 – 78.9)	355
quintuple	0	353	1.2 (0.3 – 4.6)	168	0.9 (0.3 – 2.6)	336
