# Supplementary material for: Increase in the prevalence of mutations associated with sulfadoxine–pyrimethamine resistance in Plasmodium falciparum isolates collected from early to late pregnancy in Nanoro, Burkina Faso
Source: Malar J. 2017 Apr 28;16:179. doi: 10.1186/s12936-017-1831-y (PMC5410088; doi:10.1186/s12936-017-1831-y)
Supplement: Supplementary file 2 — Additional file 2. Univariate mixed-effects logistic regression for dhfr and dhps mutations in P. falciparum positive samples. [file 12936_2017_1831_MOESM2_ESM.pdf]

Table S2. Univariate mixed-effects logistic regression for *dhfr* and *dhps* mutations in *P. falciparum* positive samples

Odds ratios (OR) with 95% CI and *p* values are presented of univariate models (*p* values <0.05 in bold).

dhfr		**N51**				**C59**				**S108**				**triple *dhfr***			
Fixed effect(s)	Samples	OR	[95% CI]		*p*	OR	[95%CI]		*p*	OR	[95%CI]		*p*	OR	[95%CI]		*p*
Age (per 10 years)	ANC	0.59	0.42	0.84	**0.004**	0.70	0.48	1.01	0.058	0.78	0.54	1.15	0.213	0.61	0.43	0.86	**0.005**
	Del	1.32	0.80	2.17	0.279	1.75	0.92	3.33	0.087	2.30	1.09	4.83	**0.028**	1.32	0.81	2.15	0.265
	GP	1.01	0.86	1.18	0.949	0.89	0.76	1.05	0.175	0.85	0.72	1.02	0.079	1.03	0.88	1.20	0.729
Parasitaemia (log scale)	ANC	1.20	0.95	1.53	0.133	1.01	0.78	1.30	0.965	1.02	0.79	1.33	0.859	1.20	0.94	1.52	0.136
	Del	0.80	0.61	1.03	0.086	0.84	0.61	1.15	0.280	0.72	0.51	1.03	0.073	0.83	0.64	1.07	0.154
	GP	1.16	0.89	1.52	0.270	1.17	0.88	1.57	0.278	1.27	0.92	1.75	0.148	1.15	0.90	1.49	0.269
Gravidity	ANC	0.92	0.83	1.02	0.100	0.93	0.84	1.04	0.212	0.97	0.86	1.08	0.569	0.91	0.82	1.01	0.075
	Del	1.20	1.03	1.41	**0.021**	1.27	1.04	1.56	**0.020**	1.39	1.09	1.77	**0.009**	1.20	1.03	1.40	**0.019**
Season#	ANC	1.00	0.66	1.52	0.990	1.01	0.64	1.57	0.975	1.00	0.63	1.59	0.994	0.98	0.65	1.48	0.926
	Del	3.98	1.67	9.53	**0.002**	4.98	1.86	13.37	**0.001**	5.42	2.05	14.29	**0.001**	4.37	1.77	10.8	**0.001**
SP doses	Del	1.01	0.71	1.42	0.966	1.26	0.80	2.00	0.320	1.41	0.83	2.41	0.204	0.97	0.88	0.70	0.881
AL	Del	0.33	0.17	0.64	**0.001**	0.59	0.27	1.30	0.190	0.61	0.26	1.45	0.268	0.37	0.19	0.70	**0.002**
Visit	ANC & Del*	1.54	0.97	2.47	0.069	2.09	1.17	3.75	**0.013**	2.64	1.20	5.81	**0.016**	1.52	1.04	2.21	**0.029**
	ANC & GP**	1.93	1.39	2.68	**0.000**	1.95	1.37	2.79	**0.000**	2.22	1.52	3.25	**0.000**	1.79	1.30	2.45	**0.000**
	GP & Del***	0.76	0.50	1.15	0.201	1.02	0.63	1.64	0.950	1.01	0.60	1.70	0.980	0.84	0.56	1.26	0.394

dhps		**S436**				**A437**			
Fixed effect(s)	Samples	OR	[95% CI]		*p*	OR	[95%CI]		*p*
Age (per 10 years)	ANC	1.08	0.73	1.60	0.696	0.79	0.52	1.19	0.266
	Del	1.12	0.67	1.87	0.653	0.68	0.31	1.48	0.325
	GP	0.86	0.75	0.99	**0.042**	0.91	0.77	1.08	0.284
Parasitaemia (log scale)	ANC	1.31	1.00	1.72	0.052	1.13	0.84	1.52	0.429
	Del	1.04	0.80	1.36	0.753	0.85	0.59	1.23	0.395
	GP	1.18	0.90	1.53	0.229	1.35	0.98	1.84	0.062
Gravidity	ANC	0.97	0.87	1.08	0.583	0.95	0.84	1.07	0.418
	Del	1.05	0.90	1.22	0.563	0.92	0.73	1.15	0.453
Season#	ANC	1.47	0.92	2.36	0.109	0.71	0.42	1.18	0.184
	Del	2.05	084	4.99	0.113	1.52	0.43	5.38	0.518
SP doses	Del	0.97	0.69	1.36	0.848	1.43	0.81	2.53	0.216
AL	Del	0.73	0.37	1.43	0.357	0.44	0.17	1.12	0.085
Visit	ANC & Del*	1.06	0.64	1.75	0.823	1.72	1.03	2.85	**0.037**
	ANC & GP**	1.57	1.11	2.23	**0.010**	1.44	0.98	2.12	0.066
	GP & Del***	0.69	0.46	1.05	0.086	1.17	0.68	2.01	0.568

AL = artemether-lumefantrine therapy; Del = delivery; # low transmission season = 0, high transmission season = 1; *ANC booking = 0, Delivery = 1; **ANC booking = 0, GP = 1; *** GP = 0, Del = 1
