# Supplementary material for: Increase in the prevalence of mutations associated with sulfadoxine–pyrimethamine resistance in Plasmodium falciparum isolates collected from early to late pregnancy in Nanoro, Burkina Faso
Source: Malar J. 2017 Apr 28;16:179. doi: 10.1186/s12936-017-1831-y (PMC5410088; doi:10.1186/s12936-017-1831-y)
Supplement: Supplementary file 3 — Additional file 3. Univariate mixed-effects logistic regression for low birth weight in P. falciparum positive women at delivery. [file 12936_2017_1831_MOESM3_ESM.pdf]

Table S3. Univariate mixed-effects logistic regression for low birth weight in *P. falciparum* positive women at delivery

Odds ratios (OR) with 95% CI and *p* values are presented of univariate models (*p* values <0.05 in bold).

dhfr<br>Fixed effect(s)	LBW<br>OR		[95%CI]	*p*
Triple *dhfr* mutation	0.95	0.39	2.30	0.905
Age (10 years)	0.53	0.28	0.99	**0.046**
Gravidity	0.74	0.60	0.91	**0.004**
Season#	1.54	0.49	4.89	0.463
IPTp-SP doses	0.47	0.28	0.78	**0.003**
AL	1.09	0.69	1.75	0.705

AL = artemether-lumefantrine therapy; LBW = low birth weight; # low transmission season = 0, high transmission season = 1;
