# Supplementary material for: Increase in the prevalence of mutations associated with sulfadoxine–pyrimethamine resistance in Plasmodium falciparum isolates collected from early to late pregnancy in Nanoro, Burkina Faso
Source: Malar J. 2017 Apr 28;16:179. doi: 10.1186/s12936-017-1831-y (PMC5410088; doi:10.1186/s12936-017-1831-y)
Supplement: Supplementary file 4 — Additional file 4. Multivariate mixed-effects logistic regression for dhfr and dhps mutations in pregnant women (recent IPTp-SP use). [file 12936_2017_1831_MOESM4_ESM.pdf]

Table S4. Multivariate mixed-effects logistic regression for *dhfr* and *dhps* mutations in pregnant women (recent IPTp-SP use)

Odds ratios (OR) with 95% CI and p values are presented (p values <0.05 in bold).

dhfr	**N51**				**C59**				**S108**				**Triple *dhfr***			
Fixed effects	OR	95% CI		*p*	OR	95% CI		*p*	OR	95% CI		*p*	OR	95% CI		*p*
Age (10 years)	0.30	0.12	0.75	**0.010**	0.47	0.17	1.28	0.139	0.48	0.17	1.35	0.166	0.38	0.19	0.80	**0.010**
Gravidity	0.24	0.97	1.59	0.087	1.12	0.84	1.48	0.439	1.16	0.86	1.57	0.326	1.17	0.94	1.45	0.165
Season#	1.06	0.67	1.66	0.818	1.05	0.61	1.82	0.849	1.03	0.58	1.82	0.920	1.03	0.67	1.57	0.896
Recent IPTp-SP†	2.16	0.83	5.65	0.116	1.76	0.52	5.96	0.362	2.73	0.63	11.79	0.178	1.71	0.73	4.01	0.219
AL	0.74	0.43	1.28	0.276	0.87	0.42	1.80	0.713	1.16	0.52	2.56	0.718	0.75	0.47	1.22	0.249
Visit*	0.19	0.03	1.23	0.081	0.24	0.03	2.33	0.219	0.20	0.02	2.38	0.201	0.18	0.03	0.97	**0.045**
SeasonXvisit	4.77	1.45	15.71	**0.010**	6.11	1.17	31.92	**0.032**	7.99	1.34	47.83	**0.023**	4.61	1.64	12.93	**0.004**
-Visit* in high transmission season	0.93	0.18	4.68	0.926	1.47	0.19	11.61	0.712	1.58	0.16	15.54	0.697	0.84	0.18	3.80	0.819
-Season# in Del samples	5.03	1.64	15.39	**0.005**	6.45	1.33	31.34	**0.021**	8.23	1.48	45.78	**0.016**	4.74	1.85	12.17	**0.001**
AgeXvisit	0.95	0.23	3.98	0.940	1.22	0.20	7.51	0.827	1.59	0.21	12.12	0.654	0.82	0.21	3.10	0.764
-Age in Del samples	0.29	0.08	1.10	0.068	0.58	0.11	3.07	0.518	0.77	0.12	5.040	0.786	0.31	0.10	0.96	**0.042**
GravidityXvisit	1.27	0.80	2.02	0.308	1.28	0.71	2.32	0.413	1.90	0.67	2.51	0.449	1.32	0.87	2.00	0.197
-Gravidity in Del samples	1.58	1.03	2.43	**0.038**	1.43	0.83	2.48	0.200	1.50	0.80	2.82	0.205	1.53	1.07	2.19	**0.019**

dhps	**S436**				**A437**			
Fixed effects	OR	95% CI		*p*	OR	95% CI		*p*
Age (10 years)	2.52	0.80	7.97	0.115	0.71	0.29	1.70	0.440
Gravidity	0.75	0.54	1.06	0.099	1.05	0.81	1.36	0.717
Season#	1.67	0.89	3.13	0.112	0.71	0.43	1.20	0.205
Recent IPTp-SP†	1.07	0.40	2.87	0.892	2.54	0.68	9.44	0.166
AL	0.85	0.48	1.52	0.587	0.58	0.32	1.03	0.061
Visit*	0.26	0.03	2.46	0.239	1.44	0.19	11.12	0.725
SeasonXvisit	1.45	0.39	5.49	0.581	2.52	0.62	10.26	0.197
-Visit* in high transmission season	0.38	0.05	3.13	0.365	3.63	0.50	26.67	0.204
-Season# in Del samples	2.42	0.69	8.49	0.166	1.80	0.49	6.64	0.377
AgeXvisit	0.42	0.07	2.64	0.353	0.93	0.17	5.23	0.934
-Age in Del samples	1.05	0.25	4.49	0.945	0.66	0.15	2.91	0.581
GravidityXvisit	1.34	0.76	2.34	0.313	0.92	0.55	1.54	0.745
-Gravidity in Del samples	1.00	0.65	1.56	0.987	0.96	0.62	1.51	0.869

Del = delivery; †= IPTp-SP dose in past 30 days (0 = no, 1 = yes); #low transmission season = 0, high transmission season = 1; *ANC1 = 0, Delivery = 1; age centred at 25 years
