# Supplementary material for: Increase in the prevalence of mutations associated with sulfadoxine–pyrimethamine resistance in Plasmodium falciparum isolates collected from early to late pregnancy in Nanoro, Burkina Faso
Source: Malar J. 2017 Apr 28;16:179. doi: 10.1186/s12936-017-1831-y (PMC5410088; doi:10.1186/s12936-017-1831-y)
Supplement: Supplementary file 5 — Additional file 5. Multivariate mixed-effects logistic regression for low birth weight in P. falciparum positive women at delivery. [file 12936_2017_1831_MOESM5_ESM.pdf]

Table S5. Multivariate mixed-effects logistic regression for low birth weight in *P. falciparum* positive women at delivery

Odds ratios (OR) with 95% CI and *p* values are presented of univariate models (*p* values <0.05 in bold).

dhfr	**LBW**			
Fixed effect(s)	OR	[95%CI]		*p*
Triple *dhfr* mutation	1.13	0.40	3.15	0.818
Age (10 years)	1.17	0.99	1.38	0.066
Gravidity	0.45	0.24	0.83	**0.010**
Season#	1.38	0.29	6.53	0.687
IPTp-SP doses	0.40	0.20	0.79	**0.008**
AL	0.75	0.40	1.40	0.367

AL = artemether-lumefantrine therapy; LBW = low birth weight; # low transmission season = 0, high transmission season = 1;
