# Supplementary material for: Increase in the prevalence of mutations associated with sulfadoxine–pyrimethamine resistance in Plasmodium falciparum isolates collected from early to late pregnancy in Nanoro, Burkina Faso
Source: Malar J. 2017 Apr 28;16:179. doi: 10.1186/s12936-017-1831-y (PMC5410088; doi:10.1186/s12936-017-1831-y)
Supplement: Supplementary file 6 — Additional file 6. Multivariate mixed-effects logistic regression for dhfr and dhps mutations in ANC and GP samples (sensitivity analysis). [file 12936_2017_1831_MOESM6_ESM.pdf]

Table S6. Multivariate mixed-effects logistic regression for *dhfr* and *dhps* mutations in ANC and GP samples (sensitivity analysis)

Odds ratios (OR) with 95% CI and p values are presented (p values <0.05 in bold).

| *dhfr* | **N51** | | | | **C59** | | | | **S108** | | | | **Triple *dhfr*** | | | |
|---|---|---|---|---|---|---|---|---|---|---|---|---|---|---|---|---|
| Fixed effects | OR | 95% CI | | *P* | OR | 95% CI | | *P* | OR | 95% CI | | *P* | OR | 95% CI | | *P* |
| Age (10 years) † | 0.59 | 0.41 | 0.84 | **0.004** | 0.70 | 0.48 | 1.01 | 0.057 | 0.78 | 0.53 | 1.15 | 0.213 | 0.60 | 0.42 | 0.86 | **0.005** |
| Season# | 1.06 | 0.69 | 1.62 | 0.798 | 1.05 | 0.67 | 1.64 | 0.847 | 1.02 | 0.64 | 1.63 | 0.926 | 1.03 | 0.68 | 1.57 | 0.877 |
| Visit* | 2.36 | 1.28 | 4.35 | **0.006** | 1.48 | 0.81 | 2.71 | 0.206 | 1.94 | 0.97 | 3.86 | 0.060 | 1.89 | 1.08 | 3.32 | **0.025** |
| AgeXvisit | 1.28 | 0.70 | 2.33 | 0.421 | 1.06 | 0.59 | 1.93 | 0.841 | 0.78 | 0.41 | 1.49 | 0.455 | 1.43 | 0.81 | 2.52 | 0.217 |
| -Age in GP samples | 0.75 | 0.47 | 1.22 | 0.246 | 0.74 | 0.46 | 1.18 | 0.203 | 0.61 | 0.36 | 1.03 | 0.064 | 0.86 | 0.68 | 1.57 | 0.877 |

| *dhps* | **S436** | | | | **A437** | | | |
|---|---|---|---|---|---|---|---|---|
| Fixed effects | OR | 95% CI | | *P* | OR | 95% CI | | *P* |
| Age (10 years) † | 1.03 | 0.69 | 1.53 | 0.882 | 0.81 | 0.54 | 1.23 | 0.336 |
| Season# | 1.51 | 0.93 | 2.44 | 0.092 | 0.71 | 0.43 | 1.20 | 0.205 |
| Visit* | 1.53 | 0.85 | 2.76 | 0.154 | 0.93 | 0.49 | 1.77 | 0.822 |
| AgeXvisit | 0.86 | 0.46 | 1.58 | 0.622 | 1.17 | 0.61 | 2.23 | 0.639 |
| -Age in GP samples | 0.88 | 0.55 | 1.41 | 0.603 | 0.95 | 0.58 | 1.57 | 0.854 |

† = age centred at 25 years and restricted to ages 15 - 45 years; # low transmission season = 0, high transmission season = 1; *ANC booking = 0, GP = 1;
