# Supplementary material for: Increase in the prevalence of mutations associated with sulfadoxine–pyrimethamine resistance in Plasmodium falciparum isolates collected from early to late pregnancy in Nanoro, Burkina Faso
Source: Malar J. 2017 Apr 28;16:179. doi: 10.1186/s12936-017-1831-y (PMC5410088; doi:10.1186/s12936-017-1831-y)
Supplement: Supplementary file 7 — Additional file 7. Univariate mixed effects logistic regression for dhfr and dhps gene mutations (pure mutants versus wild type/mixed). [file 12936_2017_1831_MOESM7_ESM.pdf]

Table S7. Univariate mixed effects logistic regression for *dhfr* and *dhps* gene mutations (pure mutants versus wild type/mixed)

Odds ratios (OR) with 95% CI and *p* values are presented (*p* values <0.05 in bold).

dhfr		**N51**				**C59**				**S108**				**triple *dhfr***			
Fixed effect(s)	Samples	OR	[95% CI]		*p*	OR	[95%CI]		*p*	OR	[95%CI]		*p*	OR	[95%CI]		*p*
Age	ANC	0.82	0.58	1.15	0.245	0.94	0.66	1.33	0.712	0.96	0.67	1.37	0.820	0.83	0.58	1.18	0.294
(per 10 years)	Del	1.46	0.90	2.37	0.130	1.55	0.89	2.68	0.118	1.65	0.89	3.07	0.110	1.28	0.81	2.01	0.287
	GP	1.14	1.00	1.31	**0.048**	1.04	0.91	1.19	0.591	0.99	0.87	1.13	0.890	1.18	1.03	1.35	**0.015**
Parasitaemia	ANC	1.11	0.88	1.39	0.390	0.92	0.73	1.17	0.502	0.93	0.73	1.17	0.521	1.14	0.91	1.44	0.260
(log scale)	Del	0.77	0.59	1.00	**0.047**	0.85	0.64	1.13	0.263	0.87	0.64	1.19	0.389	0.80	0.62	1.01	0.055
	GP	0.85	0.68	1.06	0.150	0.87	0.70	1.09	0.229	0.93	0.74	1.16	0.494	0.77	0.62	0.97	**0.026**
Gravidity	ANC	0.99	0.89	1.09	0.770	1.02	0.92	1.13	0.684	1.04	0.93	1.15	0.505	0.99	0.89	1.10	0.841
	Del	1.23	1.06	1.44	**0.008**	1.21	1.02	1.44	**0.027**	1.28	1.05	1.56	**0.014**	1.20	1.04	1.38	**0.011**
Season#	ANC	1.14	0.76	1.71	0.514	1.15	0.76	1.74	0.517	1.21	0.80	1.83	0.378	1.26	0.84	1.90	0.267
	Del	2.96	1.25	6.99	**0.013**	2.65	1.12	6.27	**0.026**	2.97	1.19	7.42	**0.020**	2.43	1.07	5.49	**0.033**
IPTp-SP doses	Del	1.07	0.77	1.51	0.678	1.32	0.88	1.98	0.178	1.46	0.91	2.36	0.119	1.14	0.82	1.57	0.436
AL	Del	0.72	0.48	1.08	0.108	0.83	0.53	1.29	0.406	0.70	0.43	1.12	0.137	0.75	0.51	1.11	0.150
Visit	ANC & Del*	2.43	1.68	3.50	**0.000**	2.68	1.79	4.03	**0.000**	3.11	2.04	4.75	**0.000**	2.22	1.55	3.18	**0.000**
	ANC & GP**	1.02	0.76	1.36	0.912	1.00	0.74	1.35	0.996	0.94	0.70	1.26	0.673	0.89	0.67	1.20	0.455

dhps		**S436**				**A437**			
Fixed effect(s)	Samples	OR	[95% CI]		*p*	OR	[95%CI]		*p*
Age	ANC	1.28	0.91	1.80	0.162	0.89	0.62	1.28	0.533
(per 10 years)	Del	1.13	0.69	1.86	0.619	0.79	0.39	1.58	0.501
	GP	1.01	0.89	1.15	0.844	1.21	1.05	1.40	**0.009**
Parasitaemia	ANC	1.01	0.80	1.29	0.916	0.96	0.74	1.24	0.756
(log scale)	Del	0.99	0.76	1.27	0.910	0.87	0.61	.122	0.416
	GP	0.92	0.74	1.15	0.486	0.90	0.72	1.12	0.340
Gravidity	ANC	1.04	0.94	1.15	0.479	0.99	0.89	1.10	0.880
	Del	1.02	0.88	1.18	0.757	0.99	0.81	1.22	0.948
Season#	ANC	1.51	1.00	2.30	0.051	0.75	0.48	1.17	0.204
	Del	1.43	0.57	3.58	0.446	1.17	0.35	3.96	0.800
IPTp-SP doses	Del	0.92	0.66	1.29	0.633	1.63	0.94	2.83	0.080
AL	Del	0.93	0.62	1.39	0.710	0.68	0.41	1.11	0.122
Visit	ANC & Del*	1.87	1.14	3.07	**0.014**	2.72	1.71	4.33	**0.000**
	ANC & GP**	0.76	0.57	1.02	0.071	0.74	0.54	1.01	0.057

Del = delivery; AL = artemether-lumefantrine therapy #dry season = 0, rainy season = 1; *ANC booking = 0, Delivery = 1; **ANC booking = 0, GP = 1
