# Supplementary material for: Increase in the prevalence of mutations associated with sulfadoxine–pyrimethamine resistance in Plasmodium falciparum isolates collected from early to late pregnancy in Nanoro, Burkina Faso
Source: Malar J. 2017 Apr 28;16:179. doi: 10.1186/s12936-017-1831-y (PMC5410088; doi:10.1186/s12936-017-1831-y)
Supplement: Supplementary file 8 — Additional file 8. Multivariate mixed effects logistic regression for dhfr and dhps gene mutations in pregnant women (pure mutants versus wild type/mixed). [file 12936_2017_1831_MOESM8_ESM.pdf]

Table S8. Multivariate mixed effects logistic regression for *dhfr* and *dhps* gene mutations in pregnant women (pure mutants versus wild type/mixed)

Odds ratios (OR) with 95% CI and *p* values are presented (*p* values <0.05 in bold).

dhfr	**N51**				**C59**				**S108**				**triple *dhfr***			
Fixed effect(s)	OR	[95% CI]		*p*	OR	[95%CI]		*p*	OR	[95%CI]		*p*	OR	[95%CI]		*p*
Age (10 years)	0.50	0.25	1.03	0.060	0.56	0.27	1.16	0.508	0.50	0.23	1.09	0.082	0.51	0.24	1.05	0.066
Gravidity	1.17	0.95	1.45	0.141	1.18	0.95	1.47	0.129	1.23	0.97	1.56	0.082	1.17	0.95	1.45	0.146
Season#	1.17	0.78	1.77	0.444	1.15	0.76	1.75	0.508	1.21	0.79	1.86	0.387	1.29	0.85	1.95	0.224
IPTp-SP doses	1.15	0.80	1.65	0.453	1.49	0.95	2.25	0.083	1.69	1.00	2.89	0.052	1.20	0.85	1.70	0.309
AL	0.82	0.53	1.28	0.385	0.97	0.59	1.58	0.896	0.83	0.49	1.41	0.499	0.87	0.56	1.34	0.523
Visit*	0.39	0.07	2.38	0.310	0.34	0.05	2.41	0.280	0.26	0.03	2.25	0.223	0.27	0.05	1.65	0.158
SeasonXvisit	2.38	0.89	6.34	0.083	2.61	0.93	7.33	0.069	2.97	0.93	9.47	0.066	1.91	0.71	5.13	0.201
-Visit* in high transmission season	0.94	0.18	4.76	0.938	0.88	0.15	5.25	0.893	0.78	0.11	5.67	0.808	0.52	0.10	2.61	0.429
-Season# in Del samples	2.79	1.14	6.80	**0.024**	3.00	1.17	7.72	**0.023**	3.59	1.17	10.99	**0.025**	2.46	1.00	6.05	**0.049**
AgeXvisit	0.79	0.22	2.87	0.722	1.18	0.30	4.68	0.810	1.08	0.25	4.78	0.915	0.54	0.15	1.98	0.356
-Age in Del samples	0.40	0.16	1.12	0.083	0.66	0.21	2.13	0.492	0.54	0.15	1.99	0.358	0.28	0.09	0.80	**0.018**
GravidityXvisit	1.29	0.86	1.94	0.211	1.13	0.73	1.73	0.589	1.19	0.73	1.92	0.484	1.42	0.95	2.12	0.090
-Gravidity in Del samples	1.52	1.07	2.14	**0.018**	1.33	0.92	1.93	0.134	1.46	0.95	2.26	0.088	1.66	1.18	2.33	**0.004**

dhps	**S436**				**A437**			
Fixed effect(s)	OR	[95% CI]		*p*	OR	[95%CI]		*p*
Age (10 years)	2.06	0.79	5.37	0.137	0.68	0.32	1.47	0.329
Gravidity	0.85	0.65	1.12	0.258	1.10	0.88	1.39	0.399
Season#	1.60	0.93	2.75	0.089	0.76	0.49	1.19	0.227
IPTp-SP doses	0.95	0.63	1.42	0.790	1.63	0.93	2.86	0.089
AL	0.94	0.57	1.56	0.821	0.67	0.40	1.12	0.125
Visit*	1.64	0.20	13.39	0.646	0.69	0.07	6.93	0.756
SeasonXvisit	0.90	0.27	3.05	0.867	1.79	0.46	6.90	0.398
-Visit* in high transmission season	1.47	0.21	10.21	0.694	1.24	0.15	10.15	0.840
-Season# in Del samples	1.44	0.47	4.41	0.522	1.36	0.38	4.87	0.635
AgeXvisit	0.72	0.15	3.55	0.686	0.66	0.13	3.47	0.626
-Age in Del samples	1.49	0.39	5.72	0.565	0.45	0.10	1.96	0.288
GravidityXvisit	1.06	0.66	1.70	0.814	1.08	0.64	1.80	0.780
-Gravidity in Del samples	0.90	0.60	1.35	0.621	1.19	0.75	1.88	0.464

Del = delivery; AL = artemether-lumefantrine therapy #dry season = 0, rainy season = 1; *ANC booking = 0, Delivery = 1; age centred at 25 years
