# Supplementary material for: Increase in the prevalence of mutations associated with sulfadoxine–pyrimethamine resistance in Plasmodium falciparum isolates collected from early to late pregnancy in Nanoro, Burkina Faso
Source: Malar J. 2017 Apr 28;16:179. doi: 10.1186/s12936-017-1831-y (PMC5410088; doi:10.1186/s12936-017-1831-y)
Supplement: Supplementary file 9 — Additional file 9. Multivariate mixed effects logistic regression for dhfr and dhps gene mutations in pregnant women sampled at ANC booking and the GP (pure mutants versus wild type/mixed). [file 12936_2017_1831_MOESM9_ESM.pdf]

Table S9. Multivariate mixed effects logistic regression for *dhfr* and *dhps* gene mutations in pregnant women sampled at ANC booking and the GP (pure mutants versus wild type/mixed)

Odds ratios (OR) with 95% CI and *p* values are presented (*p* values <0.05 in bold).

dhfr	**N51**				**C59**				**S108**				**triple *dhfr***			
Fixed effect(s)	OR	[95% CI]		*p*	OR	[95%CI]		*p*	OR	[95%CI]		*p*	OR	[95%CI]		*p*
Age	0.80	0.57	1.13	0.213	0.91	0.64	1.29	0.603	0.92	0.65	1.31	0.656	0.81	0.57	1.15	0.237
Season#	1.18	0.78	1.77	0.430	1.15	0.76	1.74	0.511	1.21	0.80	1.83	0.361	1.30	0.86	1.96	0.216
Visit*	1.24	0.85	1.81	0.254	1.12	0.76	1.64	0.566	1.03	0.71	1.51	0.857	1.17	0.80	1.72	0.409
VisitXage	1.42	0.98	2.06	0.062	1.15	0.79	1.67	0.472	1.08	0.75	1.56	0.685	1.46	1.00	2.12	0.050
- Age in GP samples	1.14	1.00	1.30	**0.046**	1.05	0.92	1.19	0.511	1.00	0.88	1.14	0.971	1.18	1.03	1.34	**0.014**

dhps	**S436**				**A437**			
Fixed effect(s)	OR	[95% CI]		*p*	OR	[95%CI]		*p*
Age	1.24	0.87	1.75	0.231	0.91	0.63	1.31	0.626
Season#	1.47	0.97	2.24	0.069	0.76	0.49	1.19	0.228
Visit*	0.93	0.64	1.35	0.690	0.76	0.50	1.15	0.198
VisitXage	0.82	0.56	1.18	0.286	1.32	0.89	1.96	0.161
- Age in GP samples	1.01	0.89	1.15	0.863	1.21	1.05	1.40	**0.010**

#dry season = 0, rainy season = 1; *ANC booking = 0, GP = 1; age centred at 25 years
